# Glitazones and alpha-glucosidase inhibitors as the second-line oral anti-diabetic agents added to metformin reduce cardiovascular risk in Type 2 diabetes patients: a nationwide cohort observational study

**DOI:** 10.1186/s12933-018-0663-6

**Published:** 2018-01-24

**Authors:** Cheng-Wei Chan, Chu-Leng Yu, Jiunn-Cherng Lin, Yu-Cheng Hsieh, Che-Chen Lin, Chen-Ying Hung, Cheng-Hung Li, Ying-Chieh Liao, Chu-Pin Lo, Jin-Long Huang, Ching-Heng Lin, Tsu-Juey Wu

**Affiliations:** 1 Cardiovascular Center, Taichung Veterans General Hospital, Taichung, Taiwan; 2 Department of Internal Medicine, Faculty of Medicine, Institute of Clinical Medicine, Cardiovascular Research Center, National Yang-Ming University School of Medicine, Taipei, Taiwan; 3 Department of Internal Medicine, Chiayi Branch, Taichung Veterans General Hospital, Chiayi, Taiwan; 4 Department of Financial and Computational Mathematics, Providence University, Taichung, Taiwan; 5 Department of Medical Research, Taichung Veterans General Hospital, Taichung, Taiwan; 6 Department of Internal Medicine, Hsinchu Branch, Taipei Veterans General Hospital, Hsinchu, Taiwan; 7 Department of Nutrition, Hung-Kuang University, Taichung, Taiwan

**Keywords:** Anti-diabetic agent, Cardiovascular risk, Metformin, Type 2 diabetes

## Abstract

**Objective:**

Metformin is the standard first-line drug for patients with Type 2 diabetes (T2DM). However, the optimal second-line oral anti-diabetic agent (ADA) remains unclear. We investigated the cardiovascular risk of various ADAs used as add-on medication to metformin in T2DM patients from a nationwide cohort.

**Methods:**

T2DM patients using different add-on oral ADAs after an initial metformin therapy of > 90 days were identified from the Taiwan National Health Insurance Database. Five classes of ADAs, including sulphonylureas (SU), glinides, thiazolidinediones (TZD), alpha-glucosidase inhibitors (AGI), and dipeptidyl peptidase-4 inhibitors (DPP-4I) were selected for analysis. The reference group was the SU added to metformin. Patients were excluded if aged < 20 years, had a history of stroke or acute coronary syndrome (ACS), or were receiving insulin treatment. The primary outcomes included any major adverse cardiovascular event (MACE) including ACS, ischemic/hemorrhagic stroke, and death. A Cox regression model was used to estimate the hazard ratio (HR) for MACE.

**Results:**

A total of 26,742 patients receiving their add-on drug to metformin of either SU (n = 24,277), glinides (n = 962), TZD (n = 581), AGI (n = 808), or DPP-4I (n = 114) were analyzed. After a mean follow-up duration of 6.6 ± 3.4 years, a total of 4775 MACEs occurred. Compared with the SU+metformin group (reference), the TZD+metformin (adjusted HR: 0.66; 95% CI 0.50–0.88, p = 0.004) and AGI+metformin (adjusted HR: 0.74; 95% CI 0.59–0.94, p = 0.01) groups showed a significantly lower risk of MACE.

**Conclusion:**

Both TZD and AGI, when used as an add-on drug to metformin were associated with lower MACE risk when compared with SU added to metformin in this retrospective cohort study.

*Trial registration* CE13152B-3. Registered 7 Mar, 2013, retrospectively registered

**Electronic supplementary material:**

The online version of this article (10.1186/s12933-018-0663-6) contains supplementary material, which is available to authorized users.

## Introduction

Patients with Type 2 diabetes (T2DM) have an increased risk of cardiovascular disease (CVD), which accounts for half of the causes of mortality in diabetic patients [1]. Given that incidence of T2DM is increasing worldwide, cardiovascular events associated with anti-diabetic therapy have become an important issue [2]. Based upon the beneficial effects of metformin shown in the UK Prospective Diabetes Study (UKPDS) [3, 4], metformin is currently recommended as the standard first-line drug therapy for patients with T2DM in clinical guidelines [5]. As diabetes is a progressive disease associated with a declining beta-cell function, second-line anti-diabetic agents (ADAs) will soon be added to metformin monotherapy in order to achieve the glycemic target [6]. Currently, there is no staunch evidence to correctly identify the most appropriate second-line ADA, particularly in terms of their impact on cardiovascular risk.

Although prospective randomized controlled trials (RCTs) have provided cardiovascular safety data on various ADAs including sulphonylureas (SU) [7], thiazolidinediones (TZD) [8–10], alpha-glucosidase inhibitors (AGI) [11, 12], and dipeptidyl peptidase-4 inhibitors (DPP-4I) [13–15], these trials were not designed to compare the individual ADAs as the add-on medication to baseline metformin monotherapy [16]. A landmark RCT comparing cardiovascular outcomes of SU, DPP-4I, glucagon-like peptide-1 analogues and insulin as second-line agents to metformin in newly diagnose T2DM patients is expected to be completed in 2020 [17]. Before any convincing clinical evidence becomes available, physicians might require real-world data which can elucidate on the cardiovascular risk associated with different add-on anti-diabetic medication, before they can make a clinical decision.

Several observational studies exploring cardiovascular risk associated with different second-line ADAs have generated diverse results. Ekström et al. reported that TZD and DPP-4I added to metformin was associated with both decreased mortality and cardiovascular events respectively, when compared to SU in a Swedish Diabetic Register Study [18]. In a Korean Health Insurance Review and Assessment Database Study, TZD (pioglitazone) added to metformin was associated with a decreased total CVD risk in patients with T2DM [19]. Another Korean Health Insurance Database Study showed that DPP-4I added to metformin had a lower CVD risk than SU added to metformin in T2DM patients [20]. However, Chang et al. using a Taiwan Diabetic Database, found that there were no differences in cardiovascular risk among several different add-on second-line oral ADAs, in a newly diagnosed diabetic population [21]. These discrepancies may have arisen due to studies of different populations, diverse cardiovascular outcomes employed, and variable observation durations followed. Of importance is that the observation duration for cardiovascular outcomes in these studies was short, ranging from 215 days to 5.6 years [18, 21], suggesting that the long-term cardiovascular risk of the different second-line ADAs added to metformin remains unclear. By using the Taiwan National Health Insurance Database which was implemented in 1995 [22, 23], we were able to investigate the long-term cardiovascular risk associated with different second-line ADAs. We hypothesized that TZD as the add-on medication to metformin decreases the cardiovascular risk when compared to SU. The presence of heart failure (HF) may abolish the cardiovascular benefits of TZD.

## Materials and methods

### Research database

The Taiwan National Health Insurance program was implemented in 1995. Currently, up to 99% of the Taiwanese population (~ 23 million) is enrolled in this program. The National Health Insurance Research Database includes figures regarding outpatient visits, hospital admissions, prescriptions, and disease records and is managed by the Taiwan National Health Research Institutes (NHRI). A systemic randomized sampling of patients’ data from 2000 to 2011, using a total of 1,000,000 subjects as the study population, was confirmed to be representative of the general Taiwanese population [22, 23]. The patients’ data was provided in an anonymous format, with written informed consents being waived. This study protocol was approved by the Institutional Review Board of Taichung Veterans General Hospital.

### Study population

Patients aged ≥ 20 years with a recent diagnosis of T2DM, were identified according to the International Classification of Diseases, Ninth Revision, Clinical Modification (ICD-9-CM) code 250 from 1999 to 2010. To avoid misclassification and to validate the diagnosis, T2DM was defined as three or more outpatient visits with a diabetic diagnosis code within a year, or at least one hospitalization with a diagnostic code of diabetes. The diabetic patients who initiated metformin as their first-line of treatment and used metformin monotherapy for a total duration of > 90 days were identified from the outpatient pharmacy prescription database. Metformin initiation was defined as being free of any oral ADAs or insulin injection before the first metformin prescription. According to the 2012 Taiwan Heart Failure Practical Guideline, heart failure (HF, ICD-9-CM code 428) diagnosis was subjectively judged by clinical physicians by the presence of either typical signs and symptoms of HF including fluid retention, weight gain, or objective evidence of cardiac dysfunction, or regular use of HF medications in the medical chart. Because the primary endpoints of the investigation was major adverse cardiovascular events (MACE) including acute coronary syndrome (ACS), ischemic/hemorrhagic stroke, and death, patients were excluded if they possessed a history of MI or stroke. Patients were also excluded if they had received oral ADAs other than metformin as their first-line of therapy, or received combination therapy (metformin plus other oral ADAs) as the first-line of therapy.

### Definitions of drug use and comparison groups

Prescribed second-line ADA usage information, including prescribed drug types, dosages, dates of prescription, and total number of pills dispensed, was obtained from an ambulatory and inpatient claims database. Patients were classified into 5 groups based on their second-line oral ADAs added to metformin: SU, glinides, TZD, AGI, and DPP-4I. The reference group was SU added to metformin, which is the most commonly used combination therapy in Taiwan. The date of the above regimen initiation was defined as the index date. During the study period, every person-day was classified into either current use or non-use. Current use was defined as using the second-line medication during the period between the prescription date and the ending date of drug supply. Discontinuation of drug therapy was defined as when no medication was refilled after the end date of the prescription.

### Study endpoint

The primary outcome of this study was the occurrence of major adverse cardiovascular events (MACE), which was a composite of all-cause mortality, acute coronary syndrome (ACS, ICD-9-CM: 410), and stroke (included fatal and nonfatal all stroke, ischemic stroke and hemorrhagic strokes; ICD-9-CM: 430–438). The study endpoint was defined as any events which occurred after the patients being added the second-line ADAs during the follow-up period (1999–2011).

### Covariate ascertainment

Demographic data including age and gender were recorded. Cardiovascular co-morbidities including hypertension, hyperlipidemia, ischemic heart disease, peripheral vascular disease, valvular heart disease, pulmonary disease, and renal disease were identified by the ICD-9-CM diagnostic code if the patient had at least 1 hospitalization or at least 3 consecutive outpatient visits of the above listed diseases.

### Statistical analysis

The data are presented as mean ± standard deviations (SD) for continuous variables, and proportions for categorical variables. Analysis of variance and Chi square tests were used for comparing differences in continuous and categorical variables. The MACE-free survival curves were plotted using the Kaplan–Meier method, and the statistical significance was examined by a log-rank test. Multivariable Cox proportional hazard regression models were used to identify potential confounding factors contributing to MACE occurrence (adjusted for age, gender, co-morbidities, and medications). We also performed stratified analysis to evaluate the cardiovascular outcomes in patients with or without the specific medications. The association between different second-line ADA use and the occurrence of MACE was expressed by the hazard ratio (HR) and a 95% confidence interval (CI). All statistical analyses were carried out using SAS software version 9.2 (SAS Institute, Inc., Cary, NC, USA). A p value of < 0.05 was considered statistically significant.

## Results

### Baseline characteristics

**Fig. 1:**
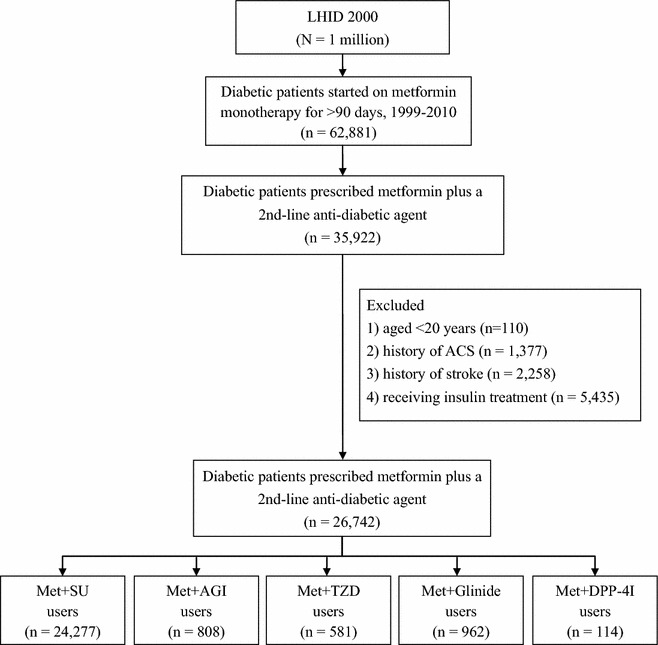
Flow chart of the study cohort. *LHID* Longitudinal Health Insurance Database, *ACS* acute coronary syndrome, *Met* metformin, *SU* sulphonylureas, *AGI* alpha-glucosidase inhibitor, *TZD* thiazolidinediones, *DPP-4I* dipeptidyl peptidase-4 inhibitor

A total of 26,742 diabetic patients were enrolled in this study. Figure 1 shows the flow chart of the study cohort. Table 1 shows the baseline characteristics of the diabetic patients receiving different second-line ADAs added to metformin. The average age of the study population was 56.4 ± 11.8 years, while 52.7% were male. The diabetic duration (metformin monotherapy duration) was 2.5 ± 2.9 years prior to adding the second-line ADA.

**Table 1:** Baseline characteristics of the diabetic patients

Variables	All patients n = 26,742	Met+SU users n = 24,277 (%)	Met+AGI users n = 808 (%)	Met+TZD users n = 581 (%)	Met+glinide users n = 962 (%)	Met+DPP-4I users n = 114 (%)	p value (5 groups)
Patient characteristics							
Age, years	56.4 (11.8)	56.3 (11.7)	56.3 (13.1)	56.4 (12.0)	57.8 (12.8)	56.2 (12.8)	0.004
Male	14,083 (52.7)	12,842 (52.9)	366 (45.3)	313 (53.9)	505 (52.5)	57 (50.0)	0.0009
Diabetes duration^a^, years	2.5 (2.9)	2.4 (2.8)	3.6 (3.5)	3.3 (3.5)	3.1 (3.5)	4.8 (4.3)	< 0.0001
Follow up duration, years	6.6 (3.4)	6.8 ± 3.4	4.7 ± 2.5	4.9 ± 2.6	5.6 ± 2.7	1.7 ± 0.5	< 0.0001
Co-morbidities							
COPD	8641 (32.3)	7770 (32.0)	299 (37.0)	194 (33.4)	329 (34.2)	49 (43.0)	0.0019
CKD	316 (1.2)	271 (1.1)	12 (1.5)	11 (1.9)	17 (1.8)	5 (4.4)	0.002
Hypertension	16,028 (60.0)	14,393 (59.3)	563 (69.7)	395 (68.0)	599 (62.3)	78 (68.4)	< 0.0001
Hyperlipidemia	15,443 (57.8)	13,794 (56.8)	589 (72.9)	404 (69.5)	571 (59.4)	85 (74.6)	< 0.0001
Heart failure^b^	1118 (4.2)	989 (4.1)	42 (5.2)	22 (3.8)	55 (5.7)	10 (8.8)	0.005
Medications							
ACEIs/ARBs	11,945 (44.7)	10,609 (43.7)	452 (55.9)	334 (57.5)	482 (50.1)	68 (59.7)	< 0.0001
Alpha blockers	3396 (12.7)	2999 (12.4)	121 (15.0)	108 (18.6)	147 (15.3)	21 (18.4)	< 0.0001
Beta blockers	13,393 (50.1)	11,966 (49.3)	516 (63.9)	326 (56.1)	513 (53.3)	72 (63.2)	<0.0001
CCB	13,034 (48.7)	11,662 (48.0)	483 (59.8)	320 (55.1)	499 (51.9)	70 (61.4)	< 0.0001
Diuretics	9434 (35.3)	8428 (34.7)	352 (43.6)	229 (39.4)	378 (39.3)	47 (41.2)	< 0.0001
Aspirin	8957 (33.5)	7939 (32.7)	348 (43.1)	246 (42.3)	376 (39.1)	48 (42.1)	< 0.0001
Clopidogrel	304 (1.1)	237 (1.0)	32 (4.0)	13 (2.2)	17 (1.8)	5 (4.4)	< 0.0001
Warfarin	176 (0.7)	152 (0.6)	10 (1.2)	4 (0.7)	10 (1.0)	0	0.11
Statins	7419 (27.7)	6408 (26.4)	359 (44.4)	270 (46.5)	325 (33.8)	57 (50.0)	< 0.0001
Fibrates	6282 (23.5)	5623 (23.2)	245 (30.3)	165 (28.4)	213 (22.1)	36 (31.6)	< 0.0001

*ACEI* angiotensin converting enzyme inhibitor, *ARB* angiotensin receptor blocker, *CCB* calcium channel blocker, *COPD* chronic obstructive pulmonary disease, *CKD* chronic kidney disease

^a^From the diagnosis of Type 2 diabetes to second-line anti-diabetic agent was add on

^b^Heart failure was judged by clinical physicians by the presence of either typical signs and symptoms of HF including fluid retention, weight gain, or objective evidence of cardiac dysfunction, or regular use of HF medications in the medical chart

Hypertension (60.0%) was the most prevalent comorbidity, followed by hyperlipidemia (57.8%) and then chronic obstructive pulmonary disease (COPD, 32.3%) in this cohort. The Met+DPP-4I group patients displayed a higher proportion of subjects with COPD (43.0%), CKD (4.4%), hyperlipidemia (74.6%) and HF (8.8%) than other groups. The proportion of patients diagnosed with hypertension was higher in the Met+AGI group (69.7%) than in other groups. Beta-blockers (50.1%) were the most frequently prescribed medications, followed by CCB (48.7%) and ACEIs/ARBSs (44.7%) in this cohort. In the Met+TZD group (n = 581), 227 patients (39.1%) used pioglitazone and 354 patients (60.9%) used rosiglitazone.

### Effects of different second-line anti-diabetic agents on cardiovascular outcomes

**Fig. 2:**
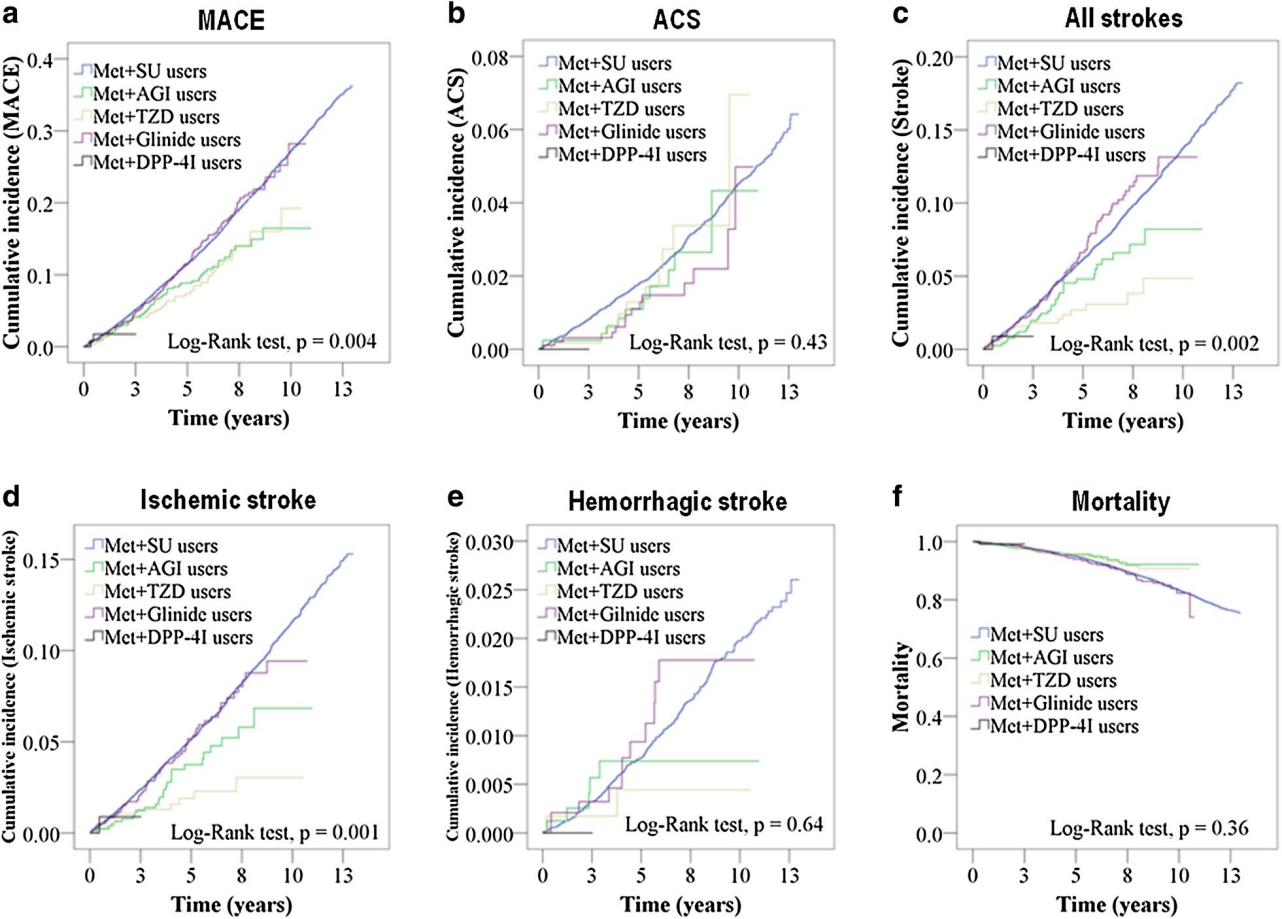
Kaplan–Meier survival curves on major adverse cardiovascular events and their composite endpoints among different second-line ADA groups. **a** major adverse cardiovascular event (MACE); **b** acute coronary syndrome (ACS); **c** all strokes; **d** ischemic stroke; **e** hemorrhagic stroke; **f** mortality

During an average of 6.6 ± 3.4 years’ follow-up, a total of 4775 MACE occurred. Table 2 shows the HRs for MACE and their composite cardiovascular endpoints. Compared to the SU group (29.0/1000 patient-years (PYs)), the incidence of MACE was significantly lower in both the TZD (17.8/1000 PYs, adjusted HR: 0.66, 95% CI 0.50–0.88, p = 0.004) and AGI (18.7/1000 PYs, adjusted HR: 0.74, 95% CI 0.59–0.94, p = 0.01) groups. There was no difference in MACE rate in patients receiving specific medications (i.e., ACEI/ARB or statin) or not among different subgroups (see Additional file 1: Table S1). In the TZD group, both pioglitazone (12.3/1000 PYs, adjusted HR: 0.54, 95% CI 0.30–0.98, p = 0.04) and rosiglitazone (20.3/1000 PYs, adjusted HR: 0.71, 95% CI 0.52–0.97, p = 0.03) groups showed a lower risk for MACE than SU (29.0/1000 PYs) group. (Additional file 1: Table S2) There was no difference in the incidence of ACS between SU and any other groups. The incidence of stroke was lower in both the TZD (56.5/1000 PYs, adjusted HR: 0.41, 95% CI 0.25–0.67, p = 0.0004) and AGI (93.3/1000 PYs, adjusted HR: 0.71, 95% CI 0.51–0.99, p = 0.04) groups than the SU (140/1000 PYs) group. The incidence of ischemic stroke was lower in both the TZD (38.7/1000 PYs, adjusted HR: 0.34, 95% CI 0.19–0.61, p = 0.0003) and AGI (71.7/1000 PYs, adjusted HR: 0.65, 95% CI 0.44–0.95, p = 0.02) groups than in the SU (117/1000 PYs) group. The incidence of hemorrhagic stroke was similar among the study groups. The incidence of all causes of mortality was also shown to be indifferent among the study groups. Figure 2 shows the Kaplan–Meier survival curves on MACE and their composite cardiovascular endpoints among different second-line ADA groups.

**Table 2:** Hazard ratios of MACE in patients receiving different 2nd-line anti-diabetic agents

Variable	Event	PYs	Rate	Crude HR (95% CI)	Adjusted HR (95% CI)	p value
MACE						
Met+SU users	4512	155,459	29.0	Ref.	Ref.	–
Met+AGI users	70	3735	18.7	0.72 (0.57–0.92)	0.74 (0.59–0.94)	0.01
Met+TZD users	50	2814	17.8	0.68 (0.51–0.89)	0.66 (0.50–0.88)	0.004
Met+glinide users	141	5187	27.2	1.01 (0.85–1.19)	0.89 (0.75–1.06)	0.18
Met+DPP-4I users	2	194	10.3	0.52 (0.13–2.08)	0.52 (0.13–2.10)	0.36
ACS						
Met+SU users	693	163,037	42.5	Ref.	Ref.	–
Met+AGI users	11	3805	28.9	0.76 (0.42–1.39)	0.74 (0.41–1.34)	0.32
Met+TZD users	10	2851	35.1	0.91 (0.49–1.71)	0.85 (0.45–1.59)	0.61
Met+glinide users	14	5364	26.1	0.66 (0.39–1.12)	0.60 (0.35–1.03)	0.06
Met+DPP-4I users	0	194	0	–	–	–
Stroke						
Met+SU users	2206	157,529	140	Ref.	Ref.	–
Met+AGI users	35	3750	93.3	0.71 (0.51–1.00)	0.71 (0.51–0.99)	0.04
Met+TZD users	16	2833	56.5	0.43 (0.26–0.70)	0.41 (0.25–0.67)	0.0004
Met+glinide users	75	5213	144	1.07 (0.85–1.35)	0.95 (0.75–1.20)	0.66
Met+DPP-4I users	1	194	51.7	0.46 (0.07–3.30)	0.46 (0.06–3.24)	0.43
Ischemic stroke						
Met+SU users	1850	158,569	117	Ref.	Ref.	–
Met+AGI users	27	3765	71.7	0.66 (0.45–0.96)	0.65 (0.44–0.95)	0.02
Met+TZD users	11	2845	38.7	0.35 (0.19–0.64)	0.34 (0.19–0.61)	0.0003
Met+glinide users	55	5247	105	0.94 (0.72–1.23)	0.83 (0.64–1.09)	0.18
Met+DPP-4I users	1	194	51.7	0.55 (0.08–3.94)	0.53 (0.07–3.77)	0.52
Hemorrhagic stroke						
Met+SU users	303	164,609	18.4	Ref.	Ref.	–
Met+AGI users	5	3813	13.1	0.77 (0.32–1.88)	0.82 (0.34–1.99)	0.66
Met+TZD users	2	2863	6.98	0.41 (0.10–1.64)	0.41 (0.10–1.64)	0.21
Met+glinide users	11	5378	20.5	1.16 (0.64–2.12)	1.08 (0.59–1.98)	0.80
Met+DPP-4I users	0	194	0	–	–	–
Mortality						
Met+SU users	2640	165,404	160	Ref.	Ref.	–
Met+AGI users	37	3824	96.8	0.78 (0.56–1.08)	0.83 (0.60–1.15)	0.26
Met+TZD users	29	2872	101	0.79 (0.55–1.14)	0.81 (0.56–1.17)	0.27
Met+glinide users	79	5392	147	1.07 (0.86–1.34)	0.93 (0.74–1.16)	0.51
Met+DPP-4I users	1	194	51.4	0.75 (0.11–5.34)	0.79 (0.11–5.61)	0.81

Multivariate Cox proportional hazards regression model was used

Model was adjusted for age, sex, diabetes duration, COPD, CKD, hypertension, heart failure, hyperlipidemia, and medications (ACEIs/ARBs, alpha blockers, beta blockers, CCB, diuretics, aspirin, clopidogrel, warfarin, statins and fibrates) used

*PYs* person-years, per 1000 PYs

### Subgroup analysis on cardiovascular outcomes in patients receiving different second-line anti-diabetic agents

Subgroup analysis comparing different second-line ADAs versus SU on the MACE incidence in diabetic patients was shown in Table 3. In patients receiving metformin plus TZD, the incidence of MACE was lower than those in the Met+SU group specifically in male (adjusted HR: 0.61, 95% CI 0.42–0.89, p = 0.01) as opposed to female (adjusted HR: 0.72, 95% CI 0.47–1.10, p = 0.13) patients. The adjusted HR for MACE was lower in both the Met+TZD (adjusted HR: 0.66, 95% CI 0.48–0.90, p = 0.009) and Met+AGI (adjusted HR: 0.77, 95% CI 0.59–1.00, p = 0.04) groups than in the Met+SU group, for patients with hypertension.

**Table 3:** Subgroup analysis of the hazard ratio for MACE in diabetic patients receiving different add-on ADAs

MACE event	Event	PYs	Rate	Crude HR (95% CI)	Adjusted HR (95% CI)	p value
< 45 years						
Met+SU users	320	27,932	115	Ref.	Ref.	–
Met+AGI users	6	708	84.7	0.94 (0.42–2.12)	0.79 (0.35–1.79)	0.57
Met+TZD users	5	479	104	1.15 (0.47–2.78)	1.04 (0.43–2.54)	0.93
Met+glinide users	8	878	91.1	0.91 (0.45–1.84)	0.99 (0.49–2.01)	0.98
Met+DPP-4I users	0	33	0	–	–	–
45–64 years						
Met+SU users	2150	93,446	230	Ref.	Ref.	–
Met+AGI users	22	2193	100	0.49 (0.32–0.74)	0.49 (0.32–0.74)	0.0009
Met+TZD users	22	1703	129	0.63 (0.41–0.95)	0.61 (0.40–0.94)	0.02
Met+glinide users	45	2890	156	0.73 (0.54–0.98)	0.72 (0.54–0.97)	0.03
Met+DPP-4I users	0	114	0	–	–	–
≥ 65 years						
Met+SU users	2042	34,081	599	Ref.	Ref.	–
Met+AGI users	42	833	504	0.96 (0.71–1.31)	1.01 (0.74–1.37)	0.96
Met+TZD users	23	632	364	0.66 (0.44–1.00)	0.65 (0.43–0.98)	0.04
Met+glinide users	88	1419	620	1.11 (0.90–1.38)	1.08 (0.87–1.33)	0.51
Met+DPP-4I users	2	47	426	0.99 (0.25–3.98)	1.04 (0.26–4.19)	0.95
Female						
Met+SU users	1934	75,370	257	Ref.	Ref.	–
Met+AGI users	35	2073	169	0.75 (0.54–1.05)	0.74 (0.53–1.03)	0.07
Met+TZD users	22	1295	170	0.74 (0.49–1.13)	0.72 (0.47–1.10)	0.13
Met+glinide users	58	2473	235	1.00 (0.77–1.30)	0.86 (0.66–1.12)	0.26
Met+DPP-4I users	2	96	208	1.23 (0.31–4.95)	1.20 (0.30–4.81)	0.80
Male						
Met+SU users	2578	80,089	322	Ref.	Ref.	–
Met+AGI users	35	1661	211	0.72 (0.52–1.01)	0.74 (0.53–1.04)	0.08
Met+TZD users	28	1518	184	0.63 (0.43–0.91)	0.61 (0.42–0.89)	0.01
Met+glinide users	83	2714	306	1.01 (0.81–1.25)	0.91 (0.73–1.14)	0.41
Met+DPP-4I users	0	97	0	–	–	
Without COPD						
Met+SU users	2783	106,567	261	Ref.	Ref.	–
Met+AGI users	34	2394	142	0.61 (0.44–0.86)	0.66 (0.47–0.93)	0.02
Met+TZD users	27	1885	143	0.61 (0.42–0.89)	0.61 (0.42–0.89)	0.01
Met+glinide users	75	3535	212	0.87 (0.69–1.09)	0.83 (0.66–1.05)	0.12
Met+DPP-4I users	1	110	90.8	0.52 (0.07–3.72)	0.50 (0.07–3.59)	0.49
With COPD						
Met+SU users	1729	48,892	354	Ref.	Ref.	–
Met+AGI users	36	1340	269	0.84 (0.61–1.17)	0.84 (0.60–1.17)	0.29
Met+TZD users	23	929	248	0.77 (0.51–1.16)	0.75 (0.49–1.13)	0.16
Met+glinide users	66	1652	400	1.22 (0.96–1.56)	0.97 (0.75–1.24)	0.78
Met+DPP-4I users	1	83	120	0.48 (0.07–3.39)	0.54 (0.08–3.85)	0.54
Without CKD						
Met+SU users	4425	153,935	287	Ref.	Ref.	–
Met+AGI users	69	3672	188	0.73 (0.58–0.93)	0.76 (0.60–0.96)	0.02
Met+TZD users	48	2780	173	0.66 (0.50–0.88)	0.65 (0.49–0.87)	0.003
Met+glinide users	135	5094	265	0.99 (0.84–1.18)	0.88 (0.74–1.05)	0.16
Met+DPP-4I users	1	185	54.0	0.28 (0.04–1.96)	0.29 (0.04–2.05)	0.21
With CKD						
Met+SU users	87	1524	571	Ref.	Ref.	–
Met+AGI users	1	63	160	0.28 (0.04–2.05)	0.31 (0.04–2.31)	0.25
Met+TZD users	2	34	594	1.28 (0.31–5.27)	1.32 (0.30–5.83)	0.72
Met+glinide users	6	93	647	1.11 (0.48–2.54)	1.06 (0.44–2.54)	0.90
Met+DPP-4I users	1	9	1175	2.60 (0.35–19.1)	2.77 (0.35–21.7)	0.33
Without hypertension						
Met+SU users	1333	67,523	197	Ref.	Ref.	–
Met+AGI users	11	1172	93.8	0.56 (0.31–1.01)	0.63 (0.34–1.14)	0.13
Met+TZD users	10	918	109	0.64 (0.34–1.19)	0.65 (0.35–1.21)	0.17
Met+glinide users	33	2106	157	0.87 (0.62–1.23)	0.85 (0.60–1.21)	0.37
Met+DPP-4I users	0	61	0	–	–	–
With hypertension						
Met+SU users	3179	87,936	362	Ref.	Ref.	–
Met+AGI users	59	2562	230	0.70 (0.54–0.91)	0.77 (0.59–1.00)	0.04
Met+TZD users	40	1896	211	0.64 (0.46–0.87)	0.66 (0.48–0.90)	0.009
Met+glinide users	108	3081	351	1.04 (0.85–1.25)	0.91 (0.75–1.10)	0.33
Met+DPP-4I users	2	132	151	0.59 (0.15–2.37)	0.63 (0.16–2.54)	0.52
Without hyperlipidemia						
Met+SU users	2321	71,328	325	Ref.	Ref.	–
Met+AGI users	23	1063	216	0.74 (0.49–1.11)	0.70 (0.46–1.05)	0.09
Met+TZD users	21	900	233	0.79 (0.51–1.21)	0.70 (0.46–1.08)	0.11
Met+glinide users	61	2249	271	0.89 (0.69–1.15)	0.83 (0.64–1.07)	0.14
Met+DPP-4I users	1	52	192	0.83 (0.12–5.87)	0.95 (0.13–6.75)	0.96
With hyperlipidemia						
Met+SU users	2191	84,131	260	Ref.	Ref.	–
Met+AGI users	47	2671	176	0.76 (0.57–1.01)	0.77 (0.58–1.03)	0.08
Met+TZD users	29	1914	152	0.64 (0.44–0.92)	0.63 (0.44–0.91)	0.01
Met+glinide users	80	2938	272	1.12 (0.90–1.40)	0.94 (0.75–1.18)	0.60
Met+DPP-4I users	1	142	70.7	0.41 (0.06–2.88)	0.37 (0.05–2.63)	0.32
Without HF						
Met+SU users	4196	150,199	279	Ref.	Ref.	–
Met+AGI users	61	3570	171	0.69 (0.54–0.89)	0.72 (0.56–0.93)	0.01
Met+TZD users	43	2732	157	0.63 (0.46–0.85)	0.61 (0.45–0.82)	0.001
Met+glinide users	128	4923	260	1.00 (0.84–1.20)	0.90 (0.75–1.07)	0.22
Met+DPP-4I users	2	174	115	0.62 (0.16–2.50)	0.67 (0.17–2.68)	0.57
With HF						
Met+SU users	316	5260	601	Ref.	Ref.	–
Met+AGI users	9	164	548	0.96 (0.50–1.87)	0.91 (0.46–1.79)	0.79
Met+TZD users	7	82	853	1.53 (0.72–3.24)	1.43 (0.67–3.04)	0.36
Met+glinide users	13	264	493	0.86 (0.49–1.50)	0.89 (0.51–1.56)	0.69
Met+DPP-4I users	0	19	0	–	–	

Multivariate Cox proportional hazards regression model was used

Model was adjusted for age, sex, diabetes duration, COPD, CKD, hypertension, heart failure, hyperlipidemia, and medications (ACEIs/ARBs, alpha blockers, beta blockers, CCB, diuretics, aspirin, clopidogrel, warfarin, statins and fibrates) used

In patients without HF, the incidence of MACE was lower in both the Met+TZD (157/1000 PYs, adjusted HR: 0.61, 95% CI 0.45–0.82, p = 0.001) and Met+AGI (171/1000 PYs, adjusted HR: 0.72, 95% CI 0.56–0.93, p = 0.01) groups than in the Met+SU (279/1000 PYs) group. However, in patients with HF, Met+TZD (853/1000 PYs, adjusted HR: 1.43, 95% CI 0.67–3.04) use was associated with an increased MACE incidence when compared to the Met+SU (601/1000 PYs) group, although the statistical significance was not reached (p = 0.36). The interaction between patients with or without HF in the Met+TZD group was significant.

## Discussion

There were two main findings in this study: (1) both TZD and AGI as add-on anti-diabetic agents to metformin reduce the risk of cardiovascular events in patients with T2DM. (2) In diabetic patients with a history of HF, add-on TZD or AGI to metformin did not reduce the risk of cardiovascular events.

### Second-line add-on anti-diabetic agents and cardiovascular risk

In clinical guidelines, metformin monotherapy is currently the standard first-line anti-diabetic therapy for patients with T2DM [5]. Given the progressive nature of T2DM, adding a second-line ADA to intensify glycemic control is unavoidable for most patients [24]. There are several classes of oral ADAs with different modes of action to control blood sugar level [25]. In addition to their efficacy for glycemic control, their impact on cardiovascular risk is of great concern to clinical physicians. Due to the lack of large RCTs to guide the most appropriate second-line ADAs, observational studies may provide the necessary real-world evidence, thus contributing to an assessment of cardiovascular risk associated with glucose-lowering therapy.

A nationwide Swedish observational study showed that when compared to SU, second-line treatment with TZD and DPP-4I as the add-on medication to metformin was associated with lower risk of mortality and cardiovascular events, respectively [18]. Seong et al. reported that when compared with a DPP-4I, TZD (pioglitazone) as the add-on medication to metformin was associated with decreased cardiovascular and ischemic stroke risk in a Korean Health Insurance Review and Assessment Database [19]. Zghebi et al. found that TZD as an add-on medication to metformin was associated with lower risk of major cardiovascular disease or death, when compared with a SU add-on treatment to metformin in an UK Clinical Practice Research Datalink [26]. Recently, a Korean Health Insurance Service Study showed that TZD as a second-line drug to metformin had relatively lower risk of CVD compared to SU, although these findings did not reach statistical significance [20]. Similar to these previous studies, we observed that both TZD and AGI as the second-line ADAs added to metformin were associated with decreased cardiovascular risk including death, stroke and ACS, although the comparators were different [18, 19, 26]. Taken together, TZD may be the most appropriate second-line medication added to metformin in patients with T2DM. However, Chang et al. in a Taiwan National Health Insurance Database Study found no differences in cardiovascular risk among several add-on second-line oral ADAs, which is contrary to not only our study, but also the above mentioned studies [21]. This discrepancy may be attributed to the differences in the inclusion criteria (metformin monotherapy for 12 months vs 90 days, respectively), diabetic duration (175–238 days vs 2.5 ± 2.9 years, respectively), composite cardiovascular outcomes (MI, heart failure, and ischemic stroke vs ACS, all stroke, and death, respectively), and observational periods (215–305 days vs 6.6 ± 3.4 years, respectively). Since cardiovascular disease was slowly progressive in T2DM patients, a long follow-up period may be essential to observe any significant outcome associated with different ADAs [4]. To the best of our knowledge, this study has undergone the longest observational duration (6.6 ± 3.4 years) among all studies comparing different ADAs as the add-on medication to metformin regarding cardiovascular outcomes.

### TZD and AGI on cardiovascular protection

In this study, we observed that both TZD and AGI as the second-line ADAs to baseline metformin reduce the risk of cardiovascular events compared to those patients using SU as their add-on medication. The reduction of MACE associated with TZD and AGI use was driven by the reduction in ischemic stroke. TZD, a potent insulin sensitizer, has favorable effects towards insulin sensitivity, plasma glucose, lipid metabolism, endothelial function, and vascular inflammation [27]. Similar to our finding, Seong et al. found that TZD (pioglitazone) plus metformin was associated with a lower risk of ischemic stroke, but not MI, when compared with the DPP-4I plus metformin group [19]. In a large scale RCT, the IRIS trial, Kernan et al. also reported that in patients with insulin resistance, the risk of stroke or myocardial infarction was lower in those using pioglitazone than a placebo [28]. Although this trial was carried out in non-diabetic patients, it has a much higher evidence level than the rest of other observational studies and proved the cardiovascular benefit for pioglitazone [28]. However, despite the fact that insulin resistance was associated with an increased risk of stroke, improving insulin sensitivity through the use of TZD did not always reduce the risk of stroke [29]. Lu et al. found that TZD (pioglitazone) did not change either cardiovascular or stroke risk when compared to the non-TZD group, among diabetic patients without macro-vascular disease [30]. The reasons why TZD did not reduce the risk of ACS in this study remains unclear. One possibility is that pioglitazone (account for 60.9% of the TZD patients) may reduce the risk of MI, while rosiglitazone (account for 39.1% of the TZD patients) may increase the MI risk in previous studies [31, 32]. Pooling both kinds of TZD users in this study might result in the neutral effect in preventing ACS comparing to SU users.

In the TZD plus metformin group, we observed that the lower incidence of MACE was observed only in male (adjusted HR: 0.61, 95% CI 0.42–0.89) in stratified analysis. This is consistent with the study conducted by Seong et al. showing that the CV risk reduction in the TZD plus metformin group was evident in male, but not female [19]. Estrogen has been shown to improve the lipid profile, increase NO signaling in the vasculature, and reduce atherosclerosis [33]. In animal study, rosiglitazone, a PPAR-γ agonist, can inhibit estrogen receptor (ER) activation and down-regulate ER expression [34]. Whether this anti-estrogen effect of TZD might accounts for the gender difference in reducing MACE by TZD remains to be explored. Further studies are needed in order to investigate the individual role of TZD in reducing the risk of stroke and MACE when it is added on to metformin.

When compared to SU, the use of AGI as the second-line ADA added to metformin decreased the risk of MACE and ischemic stroke in this study. Postprandial hyperglycemia is associated with an increase in oxidative stress, which in turn leads to endothelial dysfunction and subsequent cardiovascular diseases including ischemic stroke [35, 36]. Controlling postprandial hyperglycemia with acarbose might therefore prevent ischemic stroke [37]. The STOP-NIDDM trial showed that acarbose, a commonly used AGI in Taiwan, normalized postprandial hyperglycemia, and was also associated with a reduction in cardiovascular risk for pre-diabetic patients [11]. Consistently, acarbose has been shown to slow the progression of carotid intima-media thickness in patients diagnosed with impaired glucose tolerance, suggesting that acarbose might better prevent ischemic stroke than thrombosis at other arteries (i.e., coronary arteries) [38]. However, the Acarbose Cardiovascular Evaluation (ACE) trial, a large randomized controlled trial that unfortunately showed no cardiovascular benefit for acarbose in patients with coronary heart disease (CHD) and impaired glucose tolerance that contradicts with our result [39]. This discrepancy may be ascribed to the differences in the inclusion criteria (pre-diabetic with established CHD patients in ACE trial vs T2DM patients without CHD in this study), medication used (first-line acarbose add to cardiovascular medication vs second-line acarbose add to metformin, respectively), and composite cardiovascular outcomes (CV death, non-fatal MI, non-fatal stroke, hospital admission for unstable angina, or HF vs ACS, all stroke, and death, respectively). Therefore, the cardiovascular protective effect of acarbose as a second-line ADA to metformin has not been previously reported in diabetic patients. We provided new evidence showing that AGI as the add-on medication to metformin reduces the risk of MACE including ischemic stroke when compared to SU in diabetic patients without CHD history. Whether acarbose as a second-line medication to metformin reduces MACE risk in diabetic patients with established CHD deserved further investigation.

### Heart failure and second-line anti-diabetic medication in diabetic patients

HF occurs in 8–20% of patients with T2DM, where up to 50% of diabetic patients may develop HF during the treatment courses [40, 41]. In a national sample of medicare claims database, the mortality rates were 32.7/100 person-years in diabetic patients with HF compared with 3.7/100 person-years in diabetic patients without HF (HR 10.6, 95% CI 10.4–10.9), indicating that HF is associated with 10-times CV risk in diabetic patients [42]. TZDs, including rosiglitazone and pioglitazone, have been reported as a cause of fluid retention, while also increasing the risk of HF [31, 32, 43]. The mechanism of TZD being associated with fluid retention remains unclear, although it has been suggested that peroxisome proliferator activated receptor-gamma activation by TZD may enhance sodium channel activity in the collecting ducts and an increase in both sodium and water re-absorption and retention [44, 45]. In this study, we observed that TZD as the second-line agents was associated with a decreased cardiovascular risk when compared to SU. Subgroup analysis then showed that the cardiovascular benefit of TZD was consistent in patients without HF, indicating that TZD therapy could be favorable in patients without a history of HF. However, in patients with a history of HF, the use of TZD as the second-line agent may increase the risk of MACE (adjusted HR: 1.47, 95% CI 0.69–3.12, p = 0.32) compared to SU.

The 2017 the American Diabetes Association guideline discouraged the use of TZD as the first-line ADA in diabetic patients with HF, due to its concern of worsening HF [25]. In this study, we further found that TZD may not need to be used as a second-line ADA add-on to metformin in patients with pre-existing HF. Whether TZD as the second-line ADA to metformin monotherapy increases cardiovascular risk in diabetic patients with a history of HF deserves further investigation.

### Study strength

Previous studies comparing various ADAs added to metformin in cardiovascular outcomes were followed at a short duration [18, 19, 21, 26]. This study offered the longest observational duration (6.6 ± 3.4 years) among all the studies, and will provide robust evidence as a guideline for the appropriate second-line ADA added to metformin.

## Limitations

This study had several limitations. First, this was a non-randomized, uncontrolled observational cohort study. We could not be certain whether or not patients complied properly with their prescribed medications and dosages. Secondly, glycemic levels (evaluated by HbA1c), LDL cholesterol concentrations, kidney function (assessed by eGFR), and body mass index were not available in the Taiwan National Health Insurance Database. The degree of glycemic control and the severity of diabetes might together influence the observed CV outcome. Furthermore, low eGFR has been reported to be an independent risk factor for CV and renal events in diabetic patients [46]. Other un-available socio-demographic factors such as smoking status, physical activity, educational level, socioeconomic status, and ethnicity might also confound the CV outcome. Because of the above mentioned shortcomings, translating the study conclusions to clinical recommendations should be with cautions for specific drug therapies. Thirdly, there was a large variation in sample sizes among the different groups. Only the SU group (n = 24,277) has a large number of patients, while other groups consisted of less than 1000 patients each. Interpreting the analytical results involving these groups should be with caution. Fourth, the baseline co-morbidities (i.e., HF) of the patients in each group were not completely matched. In 2007, Nissen and Wolski raised concerns about the cardiovascular safety of rosiglitazone. This information might discourage physicians to use thiazolidinediones, either as a first-line or a second-line therapy, in diabetic patients. On the other hand, previous studies have shown that DPP-4I use was associated with a cardiovascular safety outcome, assuming DPP-4I to be a preferred choice by clinical physicians [47]. These treatment indications, clinical preference, and cost of the various drugs might contribute to the selection bias in baseline characteristics and confounded the result. However, we have adjusted the baseline co-morbidities in the analysis model to minimize this bias, making the result relevant to clinical practice. Finally, this study included mainly East Asian subjects. Whether the results could be applied to Western populations remains unknown.

## Perspectives

In recent years, several large RCTs have demonstrated cardiovascular benefits of newer glucose lowering agents not assessed in the Taiwanese cohort. By inhibiting re-absorption of urinary glucose in the proximal tubule, the sodium glucose cotransporter-2 (SGLT-2) inhibitor is a new ADA that carries a low risk for hypoglycemia. The only cardiovascular outcomes trial of SGLT-2 inhibitors to date, the EMPA-REG OUTCOME trial, showed that empagliflozin use was associated with a reduction in the primary composite endpoint of cardiovascular mortality, non-fatal MI, or non-fatal stroke compared with placebo in T2D patients [48].

There are currently 4 FDA-approved DPP-4I sitagliptin, saxagliptin, linagliptin and alogliptin under use in Taiwan. The examination of cardiovascular outcomes with alogliptin versus standard of care in patients with T2D and ACS (EXAMINE), Saxagliptin Assessment of Vascular Outcomes Recorded in Patients with Diabetes Mellitus (SAVOR-TIMI 53), and Trial Evaluating Cardiovascular Outcome with Sitagliptin (TECOS) trials have been conducted to evaluate the CV risk [47]. These DPP-4Is are safe in terms of cardiovascular endpoints [49]. However, their effect on the risk of HF remains unclear. Similarly, linagliptin use was not associated with increased cardiovascular risk a large pooled safety analysis [50]. In this study, the number of cases using DPP-4I is, only 114, too small to determine statistical difference. A new RCT comparing cardiovascular outcomes of SU, DPP-4I, glucagon-like peptide-1 analogues and insulin as second-line agents to metformin in T2DM patients is expected to be completed in 2020 [17]. Whether the above mentioned new ADAs as the second-line medication add to metformin is associated with CV safety deserved further investigation.

## Conclusion

Both TZD and AGI as add-on ADAs to metformin reduce the risk of cardiovascular events. Thus, adding a TZD or an AGI rather than a SU as a second-line agent to metformin monotherapy might be considered. In diabetic patients with a history of HF, TZD as the add-on medication did not reduce the risk of cardiovascular events.

## Additional file

**Additional file 1: Table S1.** Hazard ratios of MACE in patients receiving different 2nd-line anti-diabetic agents with or without ACEI/ARBs and statins. **Table S2.** Hazard ratios of MACE in patients receiving pioglitazone and rosiglitazone as the 2nd-line anti-diabetic agents compared to SU.

## Abbreviations

ACS: acute coronary syndrome
AGI: alpha-glucosidase inhibitor
CIs: confidence intervals
COPD: chronic obstructive pulmonary disease
CVD: cardiovascular disease
DPP-4I: dipeptidyl peptidase-4 inhibitor
ER: estrogen receptor
HF: heart failure
HR: hazard ratio
ICD-9-CM: International Classification of Diseases Ninth Revision Clinical Modification
LHID: Longitudinal Health Insurance Database
Met: metformin
NHIRD: National Health Insurance Research Database
NHRI: National Health Research Institute
OR: odds ratio
RCT: randomized controlled trial
SD: standard deviation
SU: sulphonylureas
TZD: thiazolidinediones
