# Supplementary material for: Glitazones and alpha-glucosidase inhibitors as the second-line oral anti-diabetic agents added to metformin reduce cardiovascular risk in Type 2 diabetes patients: a nationwide cohort observational study
Source: Cardiovasc Diabetol. 2018 Jan 24;17:20. doi: 10.1186/s12933-018-0663-6 (PMC5781294; doi:10.1186/s12933-018-0663-6)
Supplement: Supplementary file 1 — Additional file 1: Table S1. Hazard ratios of MACE in patients receiving different 2nd-line anti-diabetic agents with or without ACEI/ARBs and statins. Table S2. Hazard ratios of MACE in patients receiving pioglitazone and rosiglitazone as the 2nd-line anti-diabetic agents compared to SU. [file 12933_2018_663_MOESM1_ESM.docx]

Supplementary Table 1. Hazard ratios of MACE in patients receiving different 2nd-line anti-diabetic agents with or without ACEI/ARBs and statins

| MACE event | Event | PYs | Rate | Crude HR  (95% CI) | Adjusted HR  (95% CI) | p-value |
| --- | --- | --- | --- | --- | --- | --- |
| Without ACEIs/ARBs |  |  |  |  |  |  |
| Met+SU users | 2225 | 93434 | 238 | ref | ref | - |
| Met+AGI users | 29 | 1710 | 170 | 0.83(0.58-1.20) | 0.85(0.59-1.23) | 0.38 |
| Met+TZD users | 14 | 1232 | 114 | 0.55(0.32-0.93) | 0.55(0.33-0.93) | 0.03 |
| Met+Glinide users | 55 | 2763 | 199 | 0.91(0.70-1.20) | 0.86(0.65-1.12) | 0.26 |
| Met+DPP-4I users | 0 | 78 | 0 | - |  |  |
| With ACEIs/ARBs |  |  |  |  |  |  |
| Met+SU users | 2287 | 62025 | 369 | ref | ref | - |
| Met+AGI users | 41 | 2025 | 202 | 0.60(0.44-0.81) | 0.68(0.5-0.92) | 0.01 |
| Met+TZD users | 36 | 1581 | 228 | 0.66(0.48-0.92) | 0.72(0.52-1.00) | 0.05 |
| Met+Glinide users | 86 | 2424 | 355 | 1.02(0.82-1.26) | 0.91(0.74-1.13) | 0.41 |
| Met+DPP-4I users | 2 | 115 | 173 | 0.63(0.16-2.54) | 0.71(0.18-2.84) | 0.63 |
| Without statin |  |  |  |  |  |  |
| Met+SU users | 3672 | 122606 | 300 | ref | ref | - |
| Met+AGI users | 41 | 2226 | 184 | 0.69(0.50-0.93) | 0.69(0.51-0.94) | 0.02 |
| Met+TZD users | 33 | 1586 | 208 | 0.77(0.54-1.08) | 0.71(0.50-1.00) | 0.05 |
| Met+Glinide users | 106 | 3611 | 294 | 1.05(0.87-1.28) | 0.93(0.76-1.13) | 0.45 |
| Met+DPP-4I users | 1 | 99 | 101 | 0.50(0.07-3.56) | 0.55(0.08-3.90) | 0.55 |
| With statin |  |  |  |  |  |  |
| Met+SU users | 840 | 32853 | 256 | ref | ref | - |
| Met+AGI users | 29 | 1509 | 192 | 0.81(0.56-1.18) | 0.84(0.58-1.22) | 0.37 |
| Met+TZD users | 17 | 1227 | 139 | 0.57(0.35-0.93) | 0.57(0.35-0.92) | 0.02 |
| Met+Glinide users | 35 | 1576 | 222 | 0.90(0.64-1.27) | 0.79(0.56-1.11) | 0.17 |
| Met+DPP-4I users | 1 | 95 | 106 | 0.55(0.08-3.94) | 0.48(0.07-3.45) | 0.47 |

Multivariate Cox proportional hazards regression model was used.

Model was adjusted for age, sex, diabetes duration, COPD, CKD, hypertension, heart failure, hyperlipidemia, and medications (ACEIs/ARBs, alpha blockers, beta blockers, CCB, diuretics, aspirin, clopidogrel, warfarin, statins and fibrates) used.

Supplementary Table 2. Hazard ratios of MACE in patients receiving pioglitazone and rosiglitazone as the 2nd-line anti-diabetic agents compared to SU

| MACE | N | Event | PYs | Rate | Crude HR  (95% CI) | Adjusted HR  (95% CI) | p-value |
| --- | --- | --- | --- | --- | --- | --- | --- |
| Met+SU users | 24,277 | 4,512 | 155,459 | 29.0 | ref | ref | - |
| Met+pioglitazone users | 227 | 11 | 895 | 12.3 | 0.49 (0.27-0.89) | 0.54 (0.30-0.98) | 0.04 |
| Met+rosiglitazone users | 354 | 39 | 1,919 | 20.3 | 0.75 (0.55-1.03) | 0.71 (0.52-0.97) | 0.03 |

Multivariate Cox proportional hazards regression model was used.

Model was adjusted for age, sex, diabetes duration, COPD, CKD, hypertension, heart failure, hyperlipidemia, and medications (ACEIs/ARBs, alpha blockers, beta blockers, CCB, diuretics, aspirin, clopidogrel, warfarin, statins and fibrates) used.
